# Lipidic profiles of patients starting peritoneal dialysis suggest an increased cardiovascular risk beyond classical dyslipidemia biomarkers

**DOI:** 10.1038/s41598-022-20757-9

**Published:** 2022-09-30

**Authors:** Julia Hernández Lluesa, Luis Carlos López-Romero, José Jesús Broseta Monzó, Marta Roca Marugán, Iris Viejo Boyano, Diana Rodríguez-Espinosa, Aina Gómez-Bori, Amparo Soldevila Orient, Ramón Devesa Such, Pilar Sánchez Perez, Julio Hernández Jaras

**Affiliations:** 1 Metabolomics Unit, Health Research Institute Hospital La Fe (IIS La Fe), Valencia, Spain; 2 Department of Nephrology, Hospital Universitari i Politècnic La Fe, Avda. Fernando Abril Martorell 106, 46026 Valencia, Spain; 3 Department of Nephrology and Renal Transplantation, Hospital Clínic, Carrer de Villarroel 170, 08036 Barcelona, Spain

**Keywords:** Renal replacement therapy, Predictive markers, Biomarkers, Nephrology

## Abstract

Patients on peritoneal dialysis (PD) have an increased risk of cardiovascular disease (CVD) and an atherogenic lipid profile generated by exposure to high glucose dialysis solutions. In the general population, the reduction of classic lipids biomarkers is associated with improved clinical outcomes; however, the same results have not been seen in PD population, a lack of data this study aims to fulfill. Single-center prospective observational study of a cohort of CKD patients who started renal replacement therapy with continuous ambulatory peritoneal dialysis. The differences in the lipid profile and analytical variables before and 6 months after the start of peritoneal dialysis were analyzed. Samples were analyzed on an Ultra-Performance Liquid Chromatography system. Thirty-nine patients were enrolled in this study. Their mean age was 57.9 ± 16.3 years. A total of 157 endogenous lipid species of 11 lipid subclasses were identified. There were significant increases in total free fatty acids (p < 0.05), diacylglycerides (p < 0.01), triacylglycerides, (p < 0.01), phosphatidylcholines (p < 0.01), phosphatidylethanolamines (p < 0.01), ceramides (p < 0.01), sphingomyelins (p < 0.01), and cholesterol esters (p < 0.01) from baseline to 6 months. However, there were no differences in the classical lipid markers, neither lysophosphatidylcholines, monoacylglycerides, and sphingosine levels. 6 months after the start of the technique, PD patients present changes in the lipidomic profile beyond the classic markers of dyslipidemia.

## Introduction

Lipids are molecules that constitute a fundamental part of plasma and perform various cellular functions. They are mainly found as molecules of sterols, glycerophospholipids, fatty acids, and glycerolipids transported by lipoproteins (low-density lipoproteins [LDL] and high-density lipoproteins [HDL])^1^.

Phospholipids (PL) are known to be a structural part of cell membranes, perform metabolic functions and contain a variety of fatty acids such as phosphatidylserine (PS), phosphatidylethanolamine (PE), phosphatidylcholine (PC), phosphatidylinositol (PI), lysophosphatidylcholine (lysoPC) and sphingomyelin (SM)^2^. Only triglycerides (TG), LDL, HDL, and total cholesterol (TC) levels are measured in routine clinical practice as biomarkers of lipid metabolism associated with cardiovascular risk.

Patients with chronic kidney disease (CKD) have an increased risk of cardiovascular disease (CVD), to such extent that they account for most of the mortality and morbidity causes observed in this population^3^. Many other conventional risk factors for cardiovascular disease are present in patients with CKD, such as hypertension, insulin resistance, as well as underlying conditions like chronic inflammation, endothelial dysfunction, and arterial stiffness^4^. It is known that CKD produces profound changes in lipid metabolism, and these associated lipid disorders, in turn, contribute to the progression of CKD and its cardiovascular complications^5,6^.

Some authors suggest that patients on peritoneal dialysis (PD) have a more atherogenic lipid profile than non-dialysis-dependent CKD (NDD-CKD) patients or on hemodialysis (HD)^7^. This atherogenic profile is generated both by the loss of proteins in the dialysate and by the use of high glucose-based solutions^8^. Furthermore, exposure to high glucose dialysis solutions may potentially accelerate atherosclerosis and stimulate cytokines that promote chronic inflammation^9^. In the general population, the reduction of total cholesterol and LDL levels is associated with improved clinical outcomes in terms of morbidity and mortality; however, several studies and randomized clinical trials have failed to obtain these same results in patients on dialysis despite receiving treatment with lipid-lowering drugs and achieving significant reductions in LDL, CT, and TG levels^10,11^.

In recent years, the development of new technologies has produced advances in the study of the lipid profile. Lipidomics is a branch of metabolomics and a newly emerged discipline whose function is to characterize and quantify lipid molecular species and their biological role in expressing proteins involved in lipid metabolism and function^12^. It has been widely applied in disease biomarkers discovery^13^, disease mechanism research^14^, and drug development^15^. The application of these tools has demonstrated that the metabolism of phospholipids and fatty acids is modified in the context of CKD^16^.

Some metabolic networks are essential to most lipids, including sphingolipids, glycerophospholipids, and non-esterified fatty acids (NEFA). Understanding these pathways and networks will allow interpreting how and why lipids change after a specific physiological perturbation. Proton nuclear magnetic resonance spectroscopy (PNMR) and liquid chromatography–mass spectrometry (LC–MS)-based metabolomics techniques have been applied to determine CKD's metabolic features^17^. The use of lipidomics to detect a broader panel of lipid species can improve the prediction of the different alterations that influence cardiovascular mortality and the ultra-performance liquid chromatography-quadrupole time-of-flight high-definition mass spectrometry (UPLC-QTOF) is regarded as one of the best analytical tools for this purpose^18^.

Most of the numerous clinical studies focused mainly on studying classic species of lipids; however, studies of the global lipid profile in this group of patients are highly needed to comprehensively evaluate the effects in patients with end-stage chronic kidney disease.

This study aimed to analyze the lipid profile of PD patients using liquid chromatography and high-resolution mass spectrometry at the beginning and 6 months after the start of the technique.

## Results

Thirty-nine patients (26 men and 13 female) were enrolled the study. Every patient completed the 6 months follow-up. Their mean age was 57.9 ± 16.3 years. 71.8% of the patients were overweight or presented some degree of obesity with a mean body mass index of 26.72 ± 4.12 kg/m^2^. Underlying renal diseases were glomerular disease (15 patients), diabetic kidney disease (10), inherited kidney disease (4), nephroangiosclerosis (2), interstitial disease (2), and others (6). Hypertension was present in 96.3% of the patients, diabetes mellitus in 30.8%, and 30 of the 39 patients had dyslipidemia (76.9%). Of the group of patients with dyslipidaemia, only one of them was not on statin treatment.

### Evolution of classic markers of dyslipidemia

Table 1 shows the results of the analytical variables of lipid profile (TC, LDL, HDL, and TG), serum glucose, and renal function before and 6 months after initiating renal replacement therapy with CAPD. The levels of CT and LDL were lower 6 months after CAPD initiation, but these were not statistically significant. PETs from 39 patients were available. The mean D/P creatinine at 4 h was 0.76 ± 0.07.

**Table 1:** Analytical variables at baseline (T0) and 6-month onset of CAPD (T1).

	T0	T1	p value
Creatinine (mg/dl)	5.22 ± 1.06	5.81 ± 1.43	< 0.001
eGFR (ml/min/1.73 m^2^)	10.78 ± 2.13	9.60 ± 2.72	< 0.001
Glucose (mg/dl)	96.13 ± 25.86	102.43 ± 29.16	NS
Total cholesterol (mg/dl)	163.39 ± 59.96	156.91 ± 40.61	NS
LDL-C (mg/dl)	92.33 ± 50.92	84.19 ± 34.20	NS
HDL-C (mg/dl)	47.13 ± 19.50	45.08 ± 10.57	NS
Triglycerides (mg/dl)	137.17 ± 79.33	143.93 ± 82.08	NS

*eGFR* estimated glomerular filtration rate, *LDL-C* low-density lipoprotein cholesterol, *HDL* high-density lipoprotein cholesterol, *NS* statistically non-significant.

### Results of the lipidomic study

A total of 157 endogenous lipid species of 11 lipid subclasses were identified, including free fatty acids (FFA), monoacylglycerides (MAG), diacylglycerides (DAG), triacylglycerides (TAG), phosphatidylcholines (PC), lysophosphatidylcholines (LysoPC), ceramides (Cer), sphingomyelins (SM), sphingosine (SG) and cholesterol esters (CE). These results are summarized in Table 2.

**Table 2:** Lipid species identified by ultra-performance liquid chromatography (UPLC) system coupled to an iFunnel quadrupole time of flight (QTOF).

Lipid subclass	Identified compounds
Free fatty acids	26
**Glycerolipids**	
Monoacylglycerides	3
Diacylglycerides	8
Triacylglycerides	26
**Glycerophospholipids**	
Phosphatidylcholine	55
Lysophosphatidylcholine	6
Phosphatidylethanolamide	6
**Sphingolipids**	
Ceramide	3
Sphingosine	1
Sphingomyelin	19
Cholesterol esters	4
Total	157

**Figure 1:**
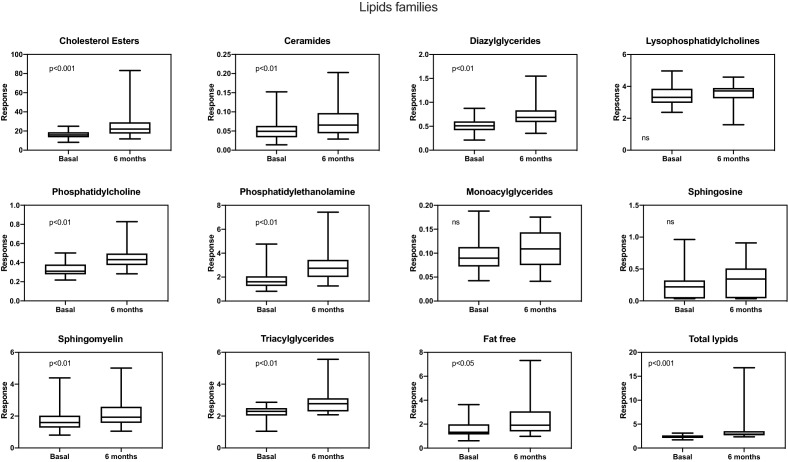
Comparison of lipid subclass levels between the measurement time at the beginning of CAPD and 6 months after.

To evaluate these findings, the majority adduct identified for each compound was selected. The differences between the two measurement times were evaluated by calculating the average data of the compounds of each group as total data. Using the plasma lipid profile, with a mass spectrometry approach, a significant increase (p < 0.05) in total free fatty acids was observed from T0 to T1. FFA (16:1) and FFA (18:0) were the FFAs with the highest peak response. In the glycerolipid group, diacylglycerides and triacylglycerides increased after initiating CAPD (p < 0.01). Monoacylglycerides had an increase but it was not statistically significant (p = 0.1). In the group of glycerophospholipids, the intensity peaks of phosphatidylcholines and phosphatidylethanolamines increased significantly (p < 0.01), but no significant changes were observed in the intensity peaks of lysophosphatidylcholines (p = 0.2). After evaluating the response of sphingolipids, we found that the response peaks of ceramides and sphingomyelins experienced a significant increase at T1 (p < 0.01). No significant changes were seen in the sphingosine response (p = 0.1). CER (18:/22:0), CER (18:1/24:0)/(18:0/24:1), and CER (18:1/24:1) were the ceramide subclasses with the highest peak response; whereas SM (18:1/16:0) and SM (42:2) were the highest among the sphingomyelin species. Cholesterol esters increased significantly at T1. CE (18:1), (18:2), and (20:4) were those that showed the most significant increase. All these results by subgroups generated a significant increase in the total lipid average responses between both studied periods. All the results analyzed by groups are summarized in Fig. 1.

## Discussion

This study aimed to evaluate the change in the main lipid class and subclass profiles after starting CAPD. Dyslipidemia is an important cardiovascular risk factor in the general population, as shown in numerous clinical studies^3,21^. The SHARP clinical study revealed these same beneficial effects in the NDD-CKD population but could not demonstrate them in those on dialysis^22^; a lack of benefit also reported in the AURORA^23^ and 4D trials^24^. Some studies have detected a paradoxical epidemiological relationship with higher mortality reported in those patients with the lowest cholesterol levels^4^; however, this could be attributed to malnourished dialysis patients with shorter life expectancy and multiple comorbidities^25^. Recently, SGLT2 inhibitors are being studied in several randomized clinical trials in peritoneal dialysis patients as a target to reduce the glucose intake in the peritoneal membrane and to improve ultrafiltration but also the cardiometabolic profile^26,27^. There is scarce data on the effect of dyslipidemia in patients in PD, and the actual value of classical lipid biomarkers is unclear.

Our LC-QTOF/MS-based lipidomics approach to study the effect of starting renal replacement therapy with CAPD in patients with CKD shows that total FFAs increased after 6 months on this technique. Within this group of lipids, FA 16:1 (palmitoleic acid) and FA 18:0 (stearic acid) were the ones that increased the most. FFAs are among the most important lipid classes and have many biological functions, regulating physiological pro-inflammatory response, dilating vessels, and signal molecules to regulate the nervous system^28,29^. A study by Friedman et al. demonstrated that FFAs levels are increased in the plasma of CKD patients; moreover, they have reported that higher levels of saturated fatty acids in serum correlate with sudden cardiac death in patients starting hemodialysis^30^. Likewise, monounsaturated fatty acids are the products of Stearoyl-CoA desaturase (SCD) catalyzed reactions and act as substrates to synthesize phospholipids and triglycerides^31^. SCD is a critical enzyme in fatty acid metabolism and an important enzyme that interacted with different hormones, such as insulin or leptin^32,33^. The activity of SCD is very high in patients with cardiovascular disease, hypertension, and diabetes^34,35^; moreover, the ratios of FFA 16:1/FFA 16:0 and FFA 18:1/FFA 18:0 levels are considered as reference values to estimate the activity of SCD^36^. Our study found that the ratios of FFA 16:1/FFA 16:0 significantly increased from T0 to T1; on the contrary, this did not happen with the ratios of FFA 18:1/FFA 18:0.

Our results show that PC and PE increased significantly at T1, but there were no changes in LysoPC levels, which is an essential component of oxidized LDL and accumulates in atherosclerotic lesions in experimental animals^37^. Therefore, it has been considered as a mediator of the atherogenic effect of oxidized LDL. LysoPCs are generated from PCs by the enzymatic activity of lecithin:cholesterol acyltransferase (LCAT). LCAT deficiency and low LysoPC levels have been reported and in HD patients, where low LysoPC levels were associated with increased cardiovascular risk compared with a control group^38^. To our knowledge, this is the first study to analyze this group of lipids in PD patients.

Alshehry et al. studies showed that sphingolipids, phospholipids, cholesteryl esters, and glycerolipids were associated with future cardiovascular events and cardiovascular death in patients with type 2 diabetes^39^. Elevated sphingomyelin levels have been associated with a risk factor for coronary heart disease and with lower survival of the PD technique^40^. To our knowledge, it is the first study to find increased levels of glycerolipid, sphingolipids, and cholesterol esters 6 months after initiating CAPD.

Our study has some limitations since it involves young patients with a short follow-up period, and unlike others that cross-compare two different groups, it has a short follow up period that made the measurement of cardiovascular events inappropriate, and as most CKD lipidomic profile studies, we only analyzed a small subset of 39 patients. On the other hand, our study’s strength is that it involves a single cohort of patients followed for 6 months with blood samples taken at baseline and after a 6-month exposure to a significant glucose load through the dialysate solutions without changes in the treatment during follow-up.

In conclusion, our results show that although the traditionally used lipid biomarkers may not change after the start of PD, the alterations in the lipidomic profile found suggest an increased cardiovascular risk in this population.

## Methods

### Study design

This is a single-center prospective observational study of a cohort of CKD patients who start renal replacement therapy with continuous ambulatory peritoneal dialysis (CAPD). This study was performed according to the ethical principles of the Declaration of Helsinki. Written informed consent was obtained from all participants before enrollment. This study is part of the project “Application of proteomics and/or metabolomics techniques for the identification and comparative analysis of uremic solutes”, approved by Hospital Universitari i Politècnic La Fe Clinical Research Ethics Committee with reference number 2014/0836.

### Study population

Data from 39 patients followed by the Department of Nephrology of Hospital Universitari i Politècnic La Fe from December 2016 to September 2018 were examined.

Subjects older than 18 years with end-stage CKD who had been seen in the clinic for at least 6 months and were programmed to start CAPD were included. Patients with malignant diseases, history of peritonitis, on treatment expecting renal function recovery, hospitalization in the previous 3 months, transferred from chronic HD, or failed kidney transplantation were excluded. The PD prescription was left at the attending physician's discretion according to the patients’ depurative and ultrafiltration requirements. All patients received commercially available glucose-based (1.36% and 2.27%) DP Dianeal solutions, and Y-sets, and twin-bag systems were applied in all cases. Of 39 patients, 34 patients (87%) started with a dialysis dose of two exchanges of 1.5 L, 1.36% glucose concentration, and a night exchange of 1.5 L of 2.27% glucose, and five (13%) patients started with an exchange of 1.5 L of 1.36% glucose concentration plus an overnight turnover of 2.27%.

### Data and laboratories collection

Baseline demographics including age, gender, major comorbidities, etiology of kidney disease, statin use, and clinical data were collected from medical records at the initiation of PD. All samples for routine laboratories and lipidomics studies were obtained the day before starting PD (T0) and 6 months after (T1). Blood samples were collected from the patients after an overnight fast and were processed immediately after sampling. Laboratory parameters included creatinine, estimated glomerular filtration rate (eGFR), glucose, TC, TG, HDL, and LDL were determined in the hospital biochemistry laboratory by assays following Good Laboratory Practice standards. Following the unit’s usual clinical practice, for the estimation of peritoneal solute transfer rate (PSTR), a standard 4-h peritoneal equilibration test (PET) with 2.27% dextrose dialysate was scheduled on T1. The peritoneal solute transfer rate was calculated using the ratio of creatinine concentration in dialysate and plasma (D/P ratio) at T1*.*

For the definition and management of dyslipidemia, the concepts and recommendations of the 2016 ESC/EAS Guidelines for the Management of Dyslipidemias, KDIGO, and ISPD guidelines were taken into account^19,41^. The KDIGO guidelines were adopted to manage dyslipidemia; thus, patients who were not receiving lipid-lowering therapy before starting the study did not begin statin therapy when starting CAPD.

### Lipidomics analysis

#### Sample preparation for mass spectrometry

Lipids were extracted by adding 150 µL of cold isopropanol to each dried sample, vortexed, and kept at − 20 °C for 30 min for protein precipitation. After centrifugation at 13,000*g* (10 min, 4 °C), 20 µL of the supernatant were transferred to a 96-well plate and 10 µL of an internal standard mix solution (IS, 20 µM), containing lipids from different classes, were added to each sample. The mixture was finally diluted with 70 µL of Mobile phase A. A quality control (QC) sample was prepared by mixing 10 µL of each sample. A blank was prepared to identify potential artifacts and analyzed at the end of the analytical sequence. Finally, samples were randomly injected into the chromatographic system in order to avoid intra-batch variability, as well as to enhance quality and reproducibility. One QC Sample was injected every five samples. Stability and analytical drift were investigated through IS intensities.

#### UPLC-QToF analysis

Samples were analyzed on an Ultra-Performance Liquid Chromatography (UPLC) system coupled to an iFunnel quadrupole time of flight (QTOF) Agilent 6550 spectrometer (Agilent Technologies, CA, USA). The chromatographic separation of lipids was carried out using an Acquity UPLC C18 CSH chromatographic column (100 × 2.1 mm, 1.8 µm) from Waters (Wexford, Milford, MA, USA). The UPLC-MS method employed was previously described by Alcoriza et al.^20^. Briefly, for ESI(+) mode, the mobile phases consisted of (A) 10 mM ammonium formate in 60:40 (v/v) acetonitrile:water and (B) 10 mM ammonium formate in 90:10 (v/v) isopropanol:acetonitrile and a flow rate of 0.4 ml/min; for ESI(−) mode, ammonium acetate was used as modifier, and the flow rate employed was 0.6 ml/min. Autosampler and column temperatures were set to 4 °C and 65 °C, respectively, and the injection volume was 5 µl.

Samples and QC were acquired using Full scan MS data from 50 to 1700 m/z with a scan frequency of 6 Hz. QC were also acquired using dependent data acquisition (DDA), by Auto MS/MS mode, and data-independent acquisition (DIA), by using the all-ion fragmentation mode, both using 0 and 40 V as collision energies.

#### Mass spectrometry data pre-processing and analysis

Data processing of samples and QC acquired in Full MS Scan was done using an in-house R (v.3.6.1) processing script with XCMS and CAMERA packages for peak detection, noise filtering, and peak alignment. QC data acquired by DIA mode were processed and annotated using the LipidMS package published by Alcoriza et al.^20^. Parameters selected for peak peaking and peak grouping were based on previous instrumental data analysis experience. The resulting data matrix was generated, including annotated molecular features, sample ID (observations), and peak intensities. Finally, an internal standard-based normalization was performed and data filtered according to the quality assurance criteria of coefficient of variation < 30% in QC samples and the presence of the variable in 60% of the samples in at least one of the compared groups. The final dataset obtained with potential lipid identification was used for the statistical analysis.

### Statistical analysis

The differences in the lipid profile analytical variables between baseline or T0 (before starting peritoneal dialysis) and T1 (6 months after the start of peritoneal dialysis) were analyzed. For the lipidomic analysis, each compound was grouped with its possible adducts and compound class based on the International Lipid Classification once the data table with the identifications had been obtained. Continuous variables were expressed as mean ± standard deviation (SD), while categorical variables were expressed as percentages. All variables were normally distributed according to the Shapiro–Wilks test and Q–Q graphs. The differences between the quantitative variables were analyzed using Student's t-test for paired data. Differences between individuals with different clinical variables were analyzed using ANOVA.

A two-sided p value inferior to 0.05 was considered statistically significant. Analyses were performed with IBM SPSS^®^ Statistics version 26 and graphics, with GraphPad version 9.

## Data Availability

The datasets used and/or analysed during the current study available from the corresponding author on reasonable request.
